# Supplementary material for: Ceg1 depletion reveals mechanisms governing degradation of non-capped RNAs in Saccharomyces cerevisiae
Source: Commun Biol. 2023 Nov 2;6:1112. doi: 10.1038/s42003-023-05495-6 (PMC10622555; doi:10.1038/s42003-023-05495-6)
Supplement: Supplementary file 2 — Supplementary Figures [file 42003_2023_5495_MOESM2_ESM.pdf]

SUPPLEMENTARY FIGURES

Supplementary Figure 1

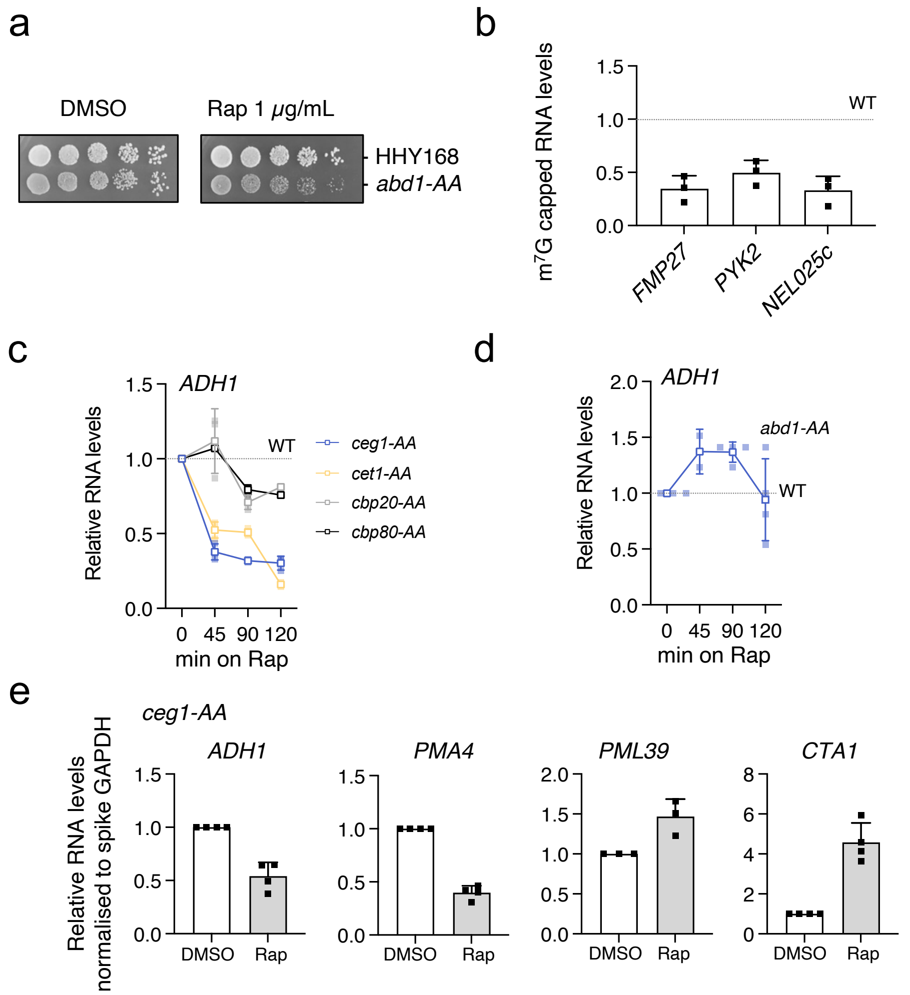

Supplementary Fig. 1:
a Spot test showing the growth of *abd1-AA* strain on DMSO and Rap in comparison with to the parental strain HHY168. Serial dilution of each strain was spotted on YPD media in the presence of DMSO or rapamycin at the final concentration of 1 μg/mL. **b** Quantification of the capped RNA by RT-qPCR after 45 min of rapamycin treatment compared to control (DMSO). The RNA isolated from yeast was spiked-in with capped human RNA and immunoprecipitated by the anti-m7G cap antibody (clone H20). The RNA levels were normalised to human *GAPDH* mRNA. The error bars show standard deviation of three independent experiments. **c** *ADH1* mRNA levels during nuclear depletion of Ceg1, Cet1, Cbp20 and Cbp80. RT-qPCR showing the RNA levels measured at 0, 45, 90 and 120 min of rapamycin treatment relative to control (DMSO). The error bars show the standard deviation of three independent experiments. **d** *ADH1* mRNA levels during nuclear depletion of Abd1 at 0, 45, 90 and 120 min of rapamycin treatment relative to control. RT-qPCR. The error bars show the standard deviation of three independent experiments. **e** Levels of selected mRNA in *ceg1-AA* growing on DMSO or Rap quantified by RT-qPCR and normalised to spike-in (human GAPDH mRNA).

Supplementary Figure 2

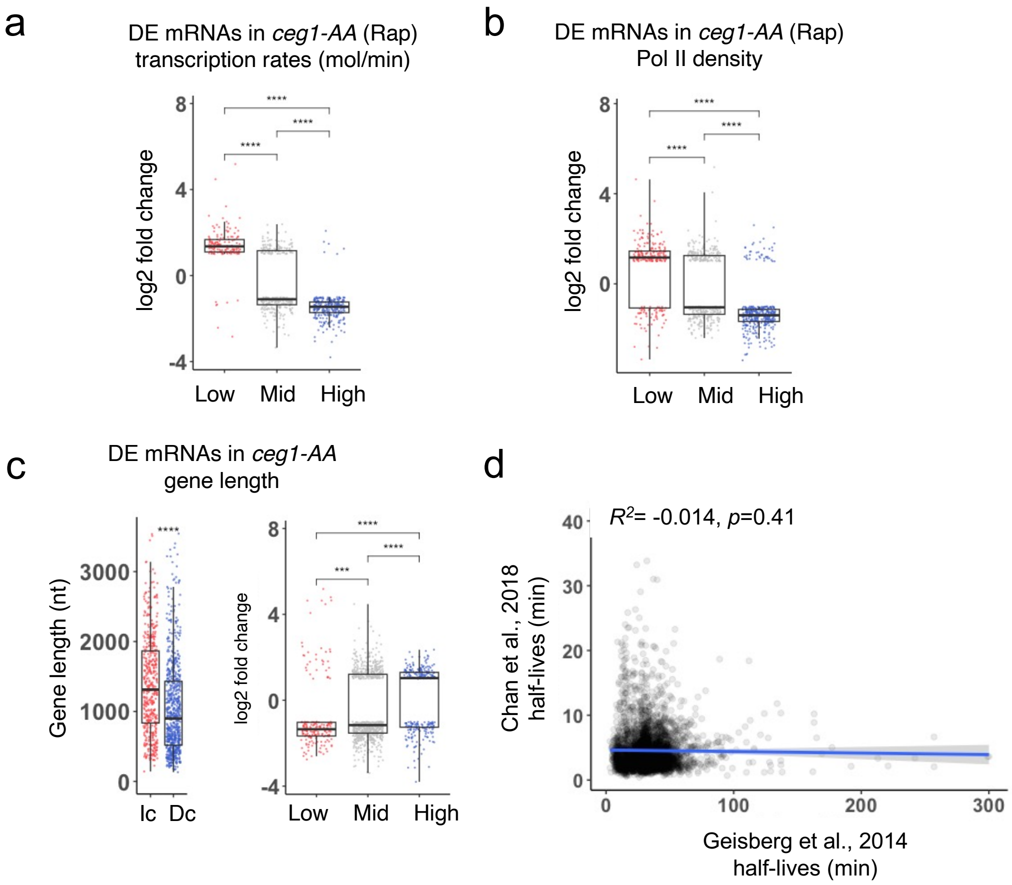

Supplementary Fig. 2:
a,b,c Fold change sorted by the expression level in *ceg1-AA* (DMSO) for differentially accumulated mRNAs in *ceg1-AA* (Rap) vs *ceg1-AA* (DMSO) plotted accordingly to their transcription rates (**a**), Pol II density (**b**) and gene length (**c**). **d** Correlation between two data sets using different methods to calculate mRNAs half-lives (Chan *et* al., 2018 and Geisberg *et* al., 2014).
The asterisks (*) indicate the statistical significance calculated via t-test or ANOVA. ns=P > 0.05; *=P ≤ 0.05; **=P ≤ 0.01; ***= P ≤ 0.001; ****= P ≤ 0.0001. The box limits show the first and the third quartile (Q1 and Q3). The line inside the box represents the median value. The lines (whisker) show the maximum and minimum value within 1.5 times interquartile Q1 and Q3. The minimum/maximum whisker values are calculated as Q1/Q3 -/+ 1.5 * IQR (interquartile range). Each point represents the value relative to a single gene.

Supplementary Figure 3

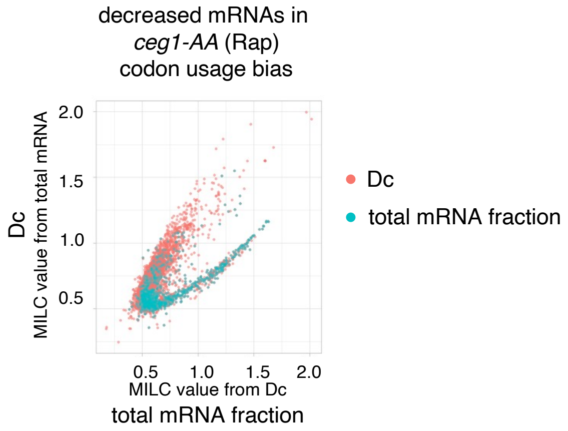

Supplementary Fig. 3:
The bias in codon usage between the total mRNA and decreased (Dc) genes differentially expressed in cells depleted of Ceg1 for 45 minutes. The plot shows Measure Independent of Length and Composition (MILC) distance between the two sets of genes.

Supplementary Figure 4

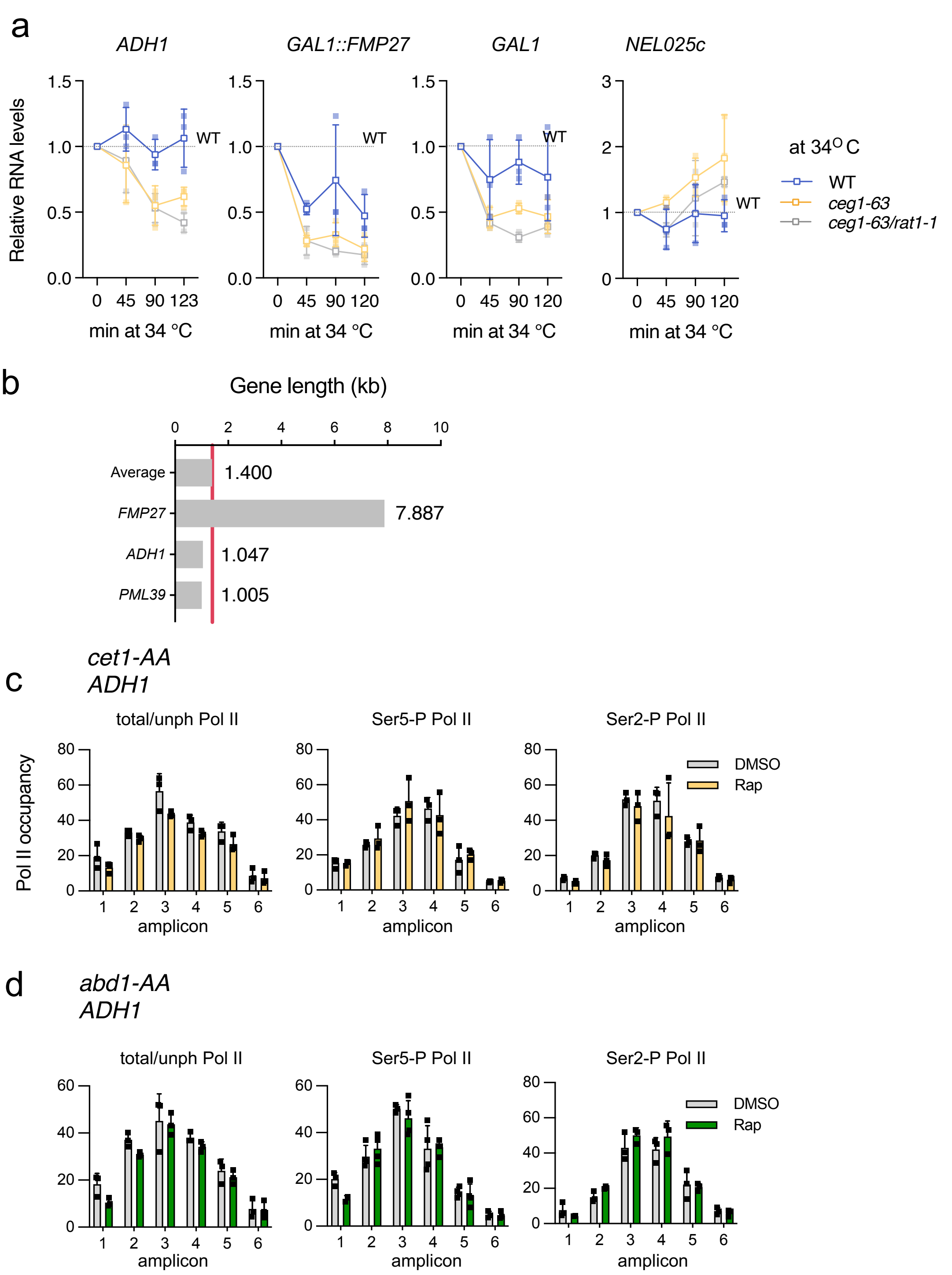

Supplementary Fig. 4:

a RNA levels of *ADH1*, *FMP27*, *GAL1* and *NEL025c* in WT and temperature-sensitive mutants *ceg1-63* and *ceg1-63/rat1-1* after 0, 45, 90 and 120 min at a sub-permissive temperature (34° C) compared to control (WT at 25° C) set to 1 (dotted lines). For analysis of *GAL1:FMP27* and *GAL1* mRNAs cells grew on galactose-containing medium. **b** Chart showing the length of FMP27, *ADH1* and *PML39* genes. The red line represents the average gene length in *S.*

cerevisiae (1.4 kb). **c,d** The distribution of total/unphosphorylated (total/unph) Pol II as well as serine 5-phosphorylated (Ser5-P) and serine 2-phosphorylated (Ser2-P) Pol II isoforms over *ADH1* in control (DMSO) and after 45 min of rapamycin (Rap) in (c) *cet1-AA* and *abd1-AA* strain (d). The location of the amplicons used for the ChIP-qPCR is shown above the charts. The error bars show the standard deviation of three independent ChIP-qPCR experiments.

Supplementary Figure 5

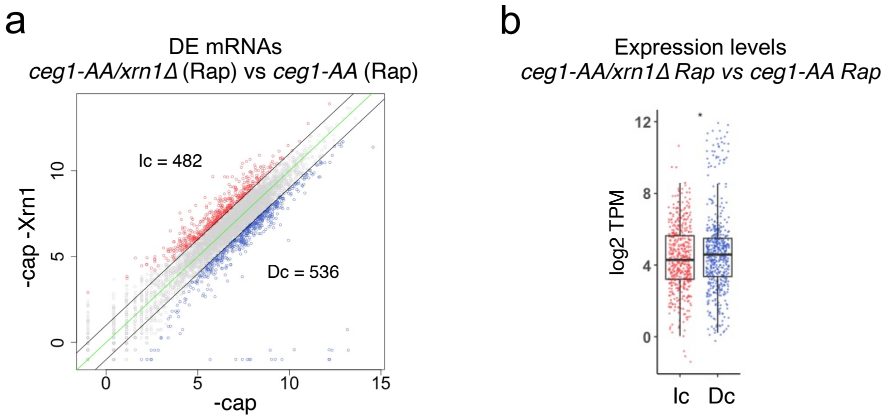

Supplementary Fig. 5:

a RNA-seq analysis showing differential accumulation of mRNA species in *ceg1-AA/xrn1Δ* (Rap) strain after 45 min of rapamycin treatment compared to *ceg1-AA* (Rap). Decreased (Dc) RNA species showing log2 Fold change <1 are in blue. Increased (Ic) RNA species with log2 Fold change >1 are labelled in red. The zero-change line is in green. **b** Differentially expressed mRNAs in *ceg1-AA/xrn1Δ* (Rap) vs *ceg1-AA* (Rap) sorted by their expression levels: Increased (Ic) or decreased (Dc). The expression levels were calculated by Transcript Per Million (TPM) value in *ceg1-AA* (DMSO). The box limits show the first and the third quartile (Q1 and Q3). The line inside the box represents the median value. The lines (whisker) show the maximum and minimum value within 1.5 times interquartile Q1 and Q3. The minimum/maximum whisker values are calculated as Q1/Q3 -/+ 1.5 * IQR (interquartile range). Each point represents the value relative to a single gene.
