# Supplementary material for: Ceg1 depletion reveals mechanisms governing degradation of non-capped RNAs in Saccharomyces cerevisiae
Source: Commun Biol. 2023 Nov 2;6:1112. doi: 10.1038/s42003-023-05495-6 (PMC10622555; doi:10.1038/s42003-023-05495-6)
Supplement: Supplementary file 3 — Description of Additional Supplementary Files [file 42003_2023_5495_MOESM3_ESM.pdf]

Description of Additional Supplementary Files

File name: Supplementary Data 1
Description: The source data behind the graphs in the paper: List of differentially expressed genes identified in RNA-seq data.

File name: Supplementary Data 2
Description: The source data behind the graphs in the paper: qPCR values

File name: Supplementary Data 3
Description: Oligonucleotides used in the study.
